# Acting against your own interests: The tension between emotion regulation preference and efficacy and its implications for individuals with depressive symptoms

**DOI:** 10.1371/journal.pone.0254213

**Published:** 2021-07-12

**Authors:** Rotem Vered, Shilat Haim-Nachum, Einat Levy-Gigi

**Affiliations:** 1 The School of Education, Bar-Ilan University, Ramat-Gan, Israel; 2 The Institute for the Study of Affective Neuroscience, University of Haifa, Haifa, Israel; 3 Department of Psychology, University of Haifa, Haifa, Israel; 4 The Gonda Multidisciplinary Brain Research Center, Bar-Ilan University, Ramat-Gan, Israel; University of Toronto at Scarborough, CANADA

## Abstract

The aim of this study was twofold: first, to compare individuals’ strategy choices in low and high intensity conditions and the actual efficacy of these strategies; second, to assess whether and how perceived intensity levels of aversive situations moderate the relationship between depressive symptoms and a strategies’ efficacy. In Experiment 1A (*N* = 58), we replicated previous results, showing that individuals prefer distraction in high- and reappraisal in low-intensity conditions, irrespective of depressive symptom levels. Experiment 1B (*N* = 50) assessed the efficacy of distraction and reappraisal strategies in aversive conditions with low and high intensity. Contrary to our prediction, reappraisal was more effective than distraction, independent of the intensity of the aversive conditions. In Experiment 2 (*N* = 113), we tested the interactive relationship between perceived intensity levels and depression on the relative effectiveness of reappraisal and distraction. We found that while in perceived low-intensity situations the advantage of distraction over reappraisal increased as depressive symptoms increased, no such relationship was found in high-intensity situations. The results suggest that while all individuals prefer to apply reappraisal in both low- and high-intensity conditions, for those with high level of depressive symptoms, such a preference acts against their own interests. The study highlights the need to distinguish between emotion regulation preferences and their actual efficacy, while illuminating possible implications for individuals with depressive symptoms.

## Introduction

Do we always choose what is right for us? Modern society supports consumer sovereignty, free marriage and democratic elections, all of which reflect the fundamental assumption that individuals are able to choose what is best for them. However, empirical investigations suggest that this is not always the case; in many instances, individuals fail to accurately predict which option will provide them with the best result [1–4]. For example, when individuals were asked to predict the pleasure they would receive from eating ice cream, there was no correlation between their predictions and their actual experience [5]. However, studies on emotion regulation tend to assume that individuals choose emotion regulation strategies that serve them the best [6, 7]. The first aim of the present study was to assess both regulatory preferences and their actual efficacy in reducing distress. The second aim was to test the effectiveness of different regulatory strategies as a function of intensity level and depressive symptoms.

Emotion regulation is defined as a range of activities that allow individuals to monitor, evaluate, and modify the nature and course of emotional responses in order to react appropriately to environmental demands [8, 9]. Two types of regulatory strategies that have been extensively studied are disengagement strategies, such as distraction, and engagement strategies, such as cognitive reappraisal. Distraction involves directing attention away from emotional information by producing neutral thoughts before an initial appraisal has been made. Reappraisal involves attending to emotional information and reinterpreting its negative meaning in order to reduce distress after an initial appraisal has been made [8, 10, 11].

According to the traditional approach, engagement strategies have been considered more effective in downregulating distress compared to disengagement strategies [12–16]. However, a more recent approach suggests that different regulatory strategies are adaptive depending on the context in which they are applied [10, 17–22]. For example, in conditions of high levels of peer victimization, habitual reappraisal is adaptive, while in conditions of low levels of peer victimization, it predicts maladaptive physiological reactivity [23].

According to the process-specific timing hypothesis, the intensity of the regulated condition and the timing of the regulation attempt determine whether a certain strategy will be adaptive or not [24, 25]. A further examination reveals that distraction is more effective in high- compared to low-intensity conditions [6, 26], since it occurs early in the emotion regulation process and does not involve engaging with the aversive situation. Conversely, reappraisal is more effective in low- compared to high-intensity conditions, since it occurs after an emotion has gained strength and requires more cognitive resources, which are more available under low-intensity conditions [27–30].

The process-specific timing hypothesis is widely supported by studies that allow participants to choose between distraction and reappraisal in low- and high-intensity conditions [6, 29, 31, 32]. These studies reveal that when given a choice, participants prefer distraction under high-intensity situations and reappraisal under low-intensity situations. However, it is not yet clear whether these preferences reflect the effectiveness of these strategies. The aim of Experiment 1A was to test distraction and reappraisal preferences in low and high intensity conditions, whereas the aim of Experiment 1B was to test the actual efficacy of distraction and reappraisal in these conditions.

Other factors that may influence the efficacy of different strategies include the personal characteristics and psychological state of the regulator [19, 33]. One such example is depression, which has a significant effect on perceiving and processing negative emotional events and may interrupt the adaptive emotional response to aversive situations [34–36]. The negative potentiation hypothesis posits that depressed individuals have a greater tendency to be aware of and fixate on adverse notions or elements in their lives [37–40]. Relatedly, depressive individuals are prone to excessive negative rumination–non-adaptive, constant focus on mundane negative thoughts [38, 41–43]. As such, they may benefit more from using strategies that do not require them to control their attention and thoughts and that allow them to avoid processing the aversive stimuli, since the processing itself can lead to rumination [44]. The aim of Experiment 2 was to investigate whether perceived intensity levels of emotional stimuli moderate the relationship between depressive symptoms and the efficacy of distraction and reappraisal in reducing distress.

## Experiment 1A

Experiment 1A sought to replicate previous findings and test individuals’ preferences for distraction or reappraisal in aversive conditions of low and high intensity. We therefore hypothesized that individuals would favor distraction in high-intensity conditions and reappraisal in low-intensity conditions. We further hypothesized that depressive and state/trait anxiety symptoms would not affect these preferences [32].

## Materials and methods

### Participants

We used G*Power software to determine a sufficient sample size given an alpha of 0.05, a power of 0.95, and a medium effect size (f = 0.25) [45]. We therefore recruited 58 college students (for a detailed description of the sample in all three experiments see Table 1), who participated in this experiment in exchange for course credit.

**Table 1:** Demographic characteristics of the participants in the three experiments (means and standard deviations/frequency).

	Experiment 1A (N = 58)	Experiment 1B (N = 50)	Experiment 2 (N = 113)
Variable	*M (SD)*	*M (SD)*	*M (SD)*
1. Age	25.26 (5.9)	24.88 (5.21)	25.15 (3.95)
2. Females/Males*	45/13	35/15	80/33
3. Education	13.64 (2.12)	14.28 (2.02)	13.88 (1.99)
4. Depression	7.67 (6.31)	4.96 (4.75)	10.52 (6.46)
5. State anxiety	36.98 (11.28)	33.3 (10.25)	39.35 (12)
5. Trait anxiety	37.81 (11.67)	33.14 (9.51)	39.61 (10.98)

*Note*.

*The values for Female/Male represent frequencies. Education is defined in years. Depressive symptoms range was 0–30, 0–21, and 1–35 for Experiment 1A, 1B and Experiment 2, respectively. Anxiety symptoms range was 20–64 and 21–68 in Experiment 1A, 20–58 and 21–58 in Experiment 1B, and 21–70, 22–76 in Experiment 2, for state and trait anxiety, respectively.

All participants were White Israelis with at least 12 years of education and several depressive symptoms. They were recruited through flyers posted in a university setting. Applicants were invited to participate in a screening interview, if they: (i) were 18–45 years of age; (ii) had accurate vision. To reduce confounds related to concurrent disorders, we used the Structured Clinical Interview for DSM-5 (SCID-5-CT) [46] to exclude participants with any current DSM-5 psychopathology according to the following exclusion criteria: (i) present or previous diagnosis of psychiatric disorders; (ii) danger of fatal harm to self or others; (iii) past experience of concussion or other medically relevant condition such as head injury, loss of consciousness for over ten minutes, or neurological conditions including epilepsy, multiple sclerosis, stroke or encephalitis. None of the participants met these criteria. Authorization was obtained from the Ethics Committee School of Education Bar-Ilan University. Participants read and signed a written informed consent form and were told that they could quit at any point during the experiment.

### Measures and procedure

In both of the two experiments, we used well-validated performance-based paradigms in which participants had to either choose between two strategies or to apply a given strategy. Based on previous studies that set criteria for low-and high-intensity and showed differential regulatory patterns in these two categories, we selected negative images of high and low intensity [6, 47]. In order to verify the correct use of the strategies, a random sample in each experiment was asked to type one sentence that described the way they employed the strategies in selected trials from each condition (distraction/reappraisal and low/high intensity). Participants wrote one sentence describing their strategy use following 25% of the trials. These sentences were then reviewed blind (with the trial’s focus on strategy and intensity remaining undisclosed) and coded for reappraisal and distraction by a clinical psychologist at a post-doctoral level.

The performance paradigm includes 30 images–all negative–from the International Affective Picture System (IAPS) [48]. The IAPS provides an official normative rating of arousal (1 –low; 9-high) and valance (1- very unpleasant; 9 –highly pleasant). The images are divided into two sets: 15 images with low (*M* arousal = 5.01; *M* valence = 3.41) and 15 images with high (*M* arousal = 6.12; *M* valence = 1.99) intensity. Low and high arousal images are statistically distinguishable across valence ratings (all *p*s < .05). The contents of the pictures are roughly matched across the different intensities [see 6]. The paradigm includes two phases: In the practice phase (four practice trials), participants learn to employ distraction (i.e. thinking about something that is emotionally neutral) and reappraisal (i.e. thinking about each picture in a way that reduces its negative meaning). In the following experimental phase, they freely choose between distraction and reappraisal for a total of 30 trials.

Each of these 30 trials begins by showing study participants a fixation cross. This is followed by a quick (500 ms) sample of a picture intended to provoke an emotional response. After the offset of the picture, a computer program issues instructions on the screen asking participants to choose one of two strategies—distraction or reappraisal. The picture then re-appears for an extended duration (5,000 ms) so that participants can implement their choice. To conclude the trial, participants use a Likert scale to assign a number to the degree of distress they felt, ranging from 1 = *no distress at all* to 9 = *great distress*. The order of the trials is randomized across participants yet ensures that no picture is presented in sequence.

The coder ratings of the sentences describing strategies use were in nearly perfect accord with the strategies utilized by the participants (99.3%). There were no significant differences between strategy effectiveness levels of the emotion regulation process for participants who wrote down these descriptions and participants who did not (all *F*s < 1), suggesting that typing out these descriptions did not influence strategy effectiveness levels.

Two questionnaires were used in order to test and control for possible effects of depression and anxiety: (1) The 21-item Beck Depression Inventory (BDI–II) [49] was used to assesses symptoms of depression over the two weeks–instead of the past week–prior to the experiment (internal consistency in the current study *α* = .89). Each item is measured on a scale from 0 to 3, with total scores ranging from 0 to 63; higher scores indicate greater levels of depression. (2) The State-Trait Anxiety Inventory (STAI) [50], a 40-item questionnaire (internal consistency in the current study *α* = .92 and .90, for state and trait anxiety, respectively) was used to evaluate individuals’ state and trait anxiety levels. Items for both state and trait anxiety are rated on a 4-point scale, with scores range from 20 to 80; higher scores reflect greater anxiety.

### Statistical analyses

In both Experiment 1 (A and B) and Experiment 2, statistical analyses were performed with SPSS version 25 (Chicago, IL). Descriptive analyses were used to calculate the demographic information of the sample. Pearson correlations were used to test associations between different experimental conditions. Repeated-measures ANOVA was used to test strategy choice preferences under low and high intensity conditions.

## Results and discussion

Zero-order correlations are presented in Table 2. We found no significant correlations between clinical measures (depression, anxiety) and experimental conditions (all *p*s > .05), suggesting that strategy preferences are not related to depressive and anxiety symptoms.

**Table 2:** Zero-order correlations between depression, anxiety, and the different conditions in Experiment 1A.

Variable	1	2	3	4	5	6	7
1. Depression	1						
2. State anxiety	.64***	1					
3. Trait anxiety	.77***	.85***	1				
4. Distraction choice—low	-.05	.18	.10	1			
5. Distraction choice–high	.10	.11	.16	.19	1		
6. Reappraisal choice–low	.05	-.18	-.10	-1.00***	-.19	1	
7. Reappraisal choice–high	-.10	-.11	-.16	-19	-1.00***	.19	1

N = 58

*Note*. The values for the experimental conditions represent choice percentages.

***p ≤ .*001*.

In order to test possible differences in individuals’ regulatory preferences in reducing distress, we conducted a strategy (distraction vs. reappraisal) by intensity (low vs. high) repeated-measures ANOVA. As predicted, and in accordance with previous research, we found that in low-intensity conditions, participants preferred to employ reappraisal on 77.7% of the low-intensity trials (95% CI: [72.83, 82.57]), whereas in high-intensity conditions, they preferred to employ distraction on 63.48% of the trials (95% CI: [50.78, 63.48), *F(*1, 56) = 29.08, *p* < .001, $\eta_{p}^{2}$ = .34. These preferences were not affected by depressive symptom levels, *F(*1,56) = .86, *p =* .36.

## Experiment 1B

In this experiment, we compared the actual effectiveness of reappraisal and distraction in reducing distress in aversive conditions with low and high intensity. According to the process-specific timing hypothesis [24], we predicted that distraction would reduce distress more successfully under high-intensity conditions, and reappraisal would reduce distress more successfully under low-intensity conditions.

## Materials and methods

### Participants

A total of 50 college students (see Table 1) participated in this experiment in exchange for course credit. Power analysis reveals that this sample is sufficient to detect a medium effect size, using an alpha of 0.05 and power of 0.95 All participants were White Israelis with at least 12 years of education. They were recruited exactly as detailed in Experiment 1A and under the same terms.

### Measures and procedure

Similar to Experiment 1A, participants use a Likert scale to assign a number to the degree of distress they felt, ranging from 1 = *no distress at all* to 9 = *great distress*. In order to compare the success of distraction versus reappraisal in reducing distress, we revised the task described in Experiment 1A such that participants were instructed which strategy to implement instead of freely choosing between them. We selected 20 pictures with low (*M* arousal = 4.96; *M* valence = 3.47) and 20 pictures with high (*M* arousal = 6.17; *M* valence = 1.99) intensity. Participants completed a short training phase (six trials) in which they employed distraction, reappraisal, or were simply asked to watch the pictures without making any overt response (look conditions). In the following experimental phase, each picture was randomly presented in three different conditions (distraction, reappraisal and look), for a total of 120 trials. The same picture was not presented twice in a row. Trial order was counterbalanced across participants.

The coder ratings of the sentences describing strategies use were in nearly perfect accord with the strategies utilized by the participants (98.81%). There were no significant differences between participants who wrote down these descriptions and participants who did not (all *F*s < 1), suggesting that typing out these descriptions did not influence participants’ responses.

### Statistical analyses

Repeated-measures ANOVA was used to analyze each strategies’ effectiveness scores in reducing distress. Paired samples *t*-test were utilized to examine differences in distress levels between the look and the strategy conditions. Pearson correlations were used to test associations between different experimental conditions.

## Results and discussion

We calculated six raw scores for each participant which represent the mean distress level in each of the six experimental conditions (Look/Distraction/Reappraisal X Low/High intensity). Specifically, we computed distress levels in the look conditions for both low (*M* = 399, *SD* = 153.97) and high (*M* = 655.38, *SD* = 157.56) intensity; distress levels after implementing distraction in low (*M* = 103.6, *SD* = 103.48) and high (*M* = 150.2, *SD* = 128.83) intensity conditions, or reappraisal in low (*M* = 135, *SD* = 127.71) and high (*M* = 167.8, *SD* = 140.45) intensity conditions. Preliminary paired samples *t*-tests revealed that the level of distress in the look conditions was significantly higher than the level of distress following implementation of distraction *t*(49) = 21.55, *p* < .001, CI 95% [362.96, 437.62] and reappraisal *t*(49) = 22.7, *p* < .001, CI 95% [342.53, 409.05]. The effectiveness of distraction and reappraisal was calculated by computing look trial distress minus the level of distress after the employment of the distraction and reappraisal strategies, with higher scores reflecting greater effectiveness. These measures better represented the efficacy of each strategy, since they took into account not only the levels of distress following distraction or reappraisal but also the relationship between these levels and the distress activated in the look trials. Correlations between the experimental conditions are presented in Table 3.

**Table 3:** Zero-order correlations between depression, anxiety, and the different conditions in Experiment 1B.

Variable	1	2	3	4	5	6	7	8	9
1. Depression	1								
2. State anxiety	.33*	1							
3. Trait anxiety	.49**	.79	1						
4. Look–low	.08	-.04	-.11	1					
5. Look–high	.14	-.05	-.10	.78***	1				
6. Distraction–low	.14	.06	.21	.63***	.42	1			
7. Distraction–high	.24	.10	.24	.37***	.39**	.74***	1		
8. Reappraisal–low	.05	-.08	.02	.72***	.55***	.89***	.67***	1	
9. Reappraisal–high	.04	.04	.10	.52***	.52***	.72***	.82***	.80***	1

N = 50

*Note*. The values for the experimental conditions represent mean distress scores in low and high intensity conditions.

*p < .05

**p < 01

***p ≤ .001.

**Fig 1:**
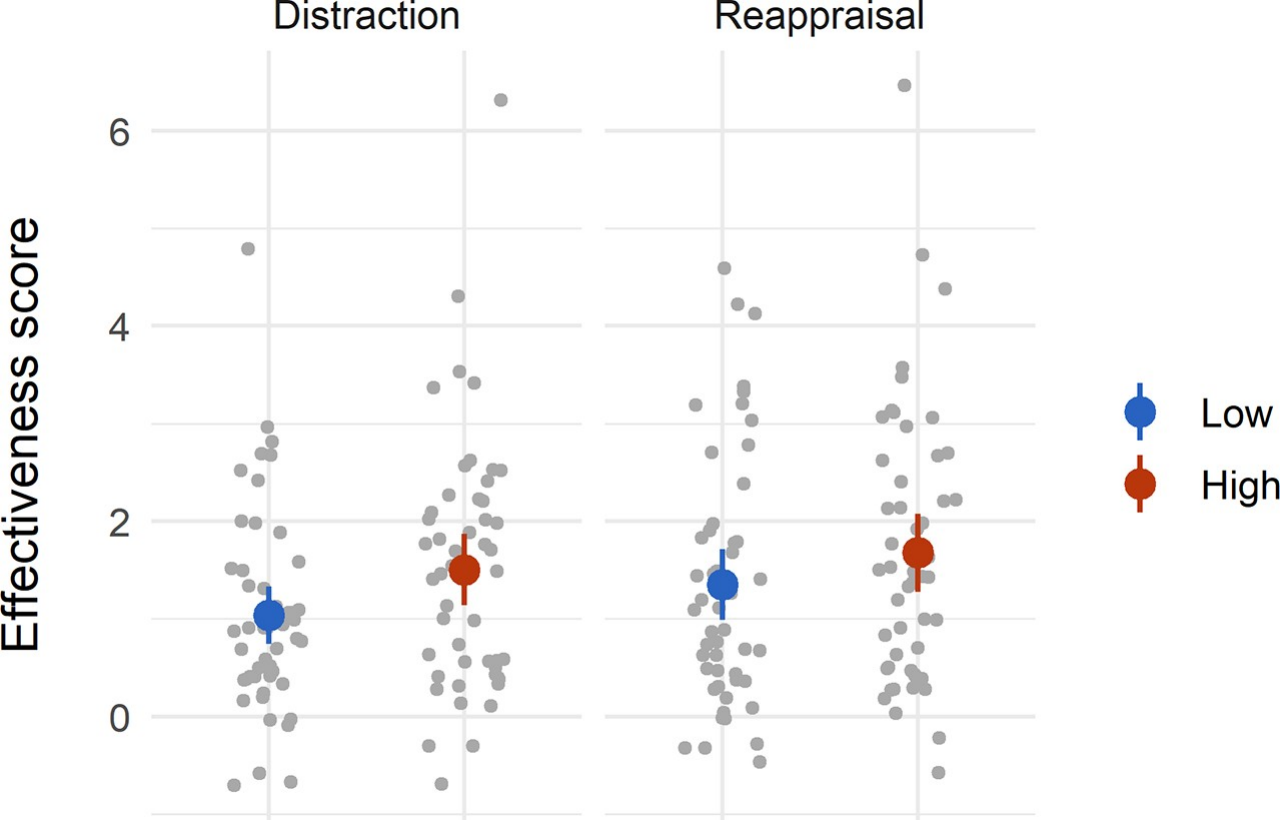
Effectiveness of regulatory strategies compared to intensity levels (Experiment 1B, N = 50). Error bars represent 95% confidence intervals (CI). Mean effectiveness scores can range between 0 to 8.

To test possible differences in the effectiveness of different regulatory strategies in reducing distress, we conducted a strategy (distraction vs. reappraisal) by intensity (low vs. high) repeated-measures ANOVA on the strategies’ effectiveness score. The results are presented in Fig 1. We predicted and found a significant main effect of intensity *F*(1,49*) =* 12.5, *p =* .001*, $\eta_{p}^{2}$ = .*20, indicating that emotion regulation attempts were more effective in high (*M =* 159, *SD =* 18.18) compared to low intensity (*M* = 119.3, *SD =* 15.9) negative images. This finding is in line with prior evidence showing that the benefit of regulatory strategies increases in conditions where they are most needed, as there is more negative emotion to regulate [26, 32]. In addition, we found a significant main effect of strategy *F(*1,49) = 7.7, *p* = .01, $\eta_{p}^{2}$ = .13, demonstrating that reappraisal (*M =* 151.4, *SD =* 18) was more effective than distraction (*M* = 126.9, *SD =* 15.34) in reducing distress. However, there was no significant interaction between intensity and strategy *F(*1,49) = 2.15, *p =* .15, indicating that the effectiveness of each strategy did not differ as a function of stimulus intensity. Hence, reappraisal was more effective in reducing distress than distraction, independent of the stimulus intensity level.

To summarize, Experiment 1 showed a discrepancy between individuals’ preferred strategies and the efficacy of those strategies in reducing distress. Specifically, while participants chose distraction in high-intensity conditions and reappraisal in low-intensity conditions, reappraisal was in fact more effective in reducing distress. In Experiment 2, we investigated individuals with subclinical depressive symptoms to test the effect of symptom level on the actual effectiveness of distraction and reappraisal.

## Experiment 2

Depression impacts individuals’ emotional reactivity and response to negative events [35, 36]. Experiment 2 sought to assess the relationship between depressive symptoms and strategies’ effectiveness and the moderating role of intensity levels in this relationship. In line with the negative potentiation hypothesis, we expected that increased intensity levels would lead to higher emotional reactivity and would thus result in the greater effectiveness of distraction compared to reappraisal in depressive individuals.

## Materials and methods

### Participants

Based on the effect size that was found in a previous related study [47], we conducted an a-priori power assessment for moderation analysis, given an alpha of 0.05, a power of 0.95, and a medium effect size (f = 0.25). The analysis revealed the need for 107 participants. The estimated sample size was increased by 5% to account for potential equipment failure and to ensure high data quality. Accordingly, we recruited 114 Israeli college students in exchange for course credit. One was excluded due to missing data for a total of 113 participants (see Table 1). Participants were prescreened and included only if they had at least one depressive symptom. Other than that, participants were recruited exactly as detailed in Experiment 1 and under the same terms.

### Measures, procedure, and analyses

We used the same paradigm detailed in Experiment 1B and the procedure was identical. Since it is not yet clear how depressed individuals perceive aversive situations, i.e., some studies suggest that they perceive them as more aversive [51, 52] while others propose that they perceive them as less aversive [40, 53], we wished to control for perceived intensity levels. Thus, we created a variable of intensity indicating the mean distress level participants reported under the “look” conditions, where participants had to simply watch aversive images and respond naturally.

Similar to Experiment 1A and 1B, the coder ratings of the sentences describing strategies use were in nearly perfect accord with the strategies utilized by the participants (98.92%). There were no significant differences between participants who wrote down these descriptions and participants who did not (all *F*s < 1), suggesting that typing out these descriptions did not influence participants’ responses. The Hayes [54] PROCESS macro, Model 1, was used to test the interactive effect of depressive symptoms and perceived intensity on strategy effectiveness.

## Results and discussion

**Fig 2:**
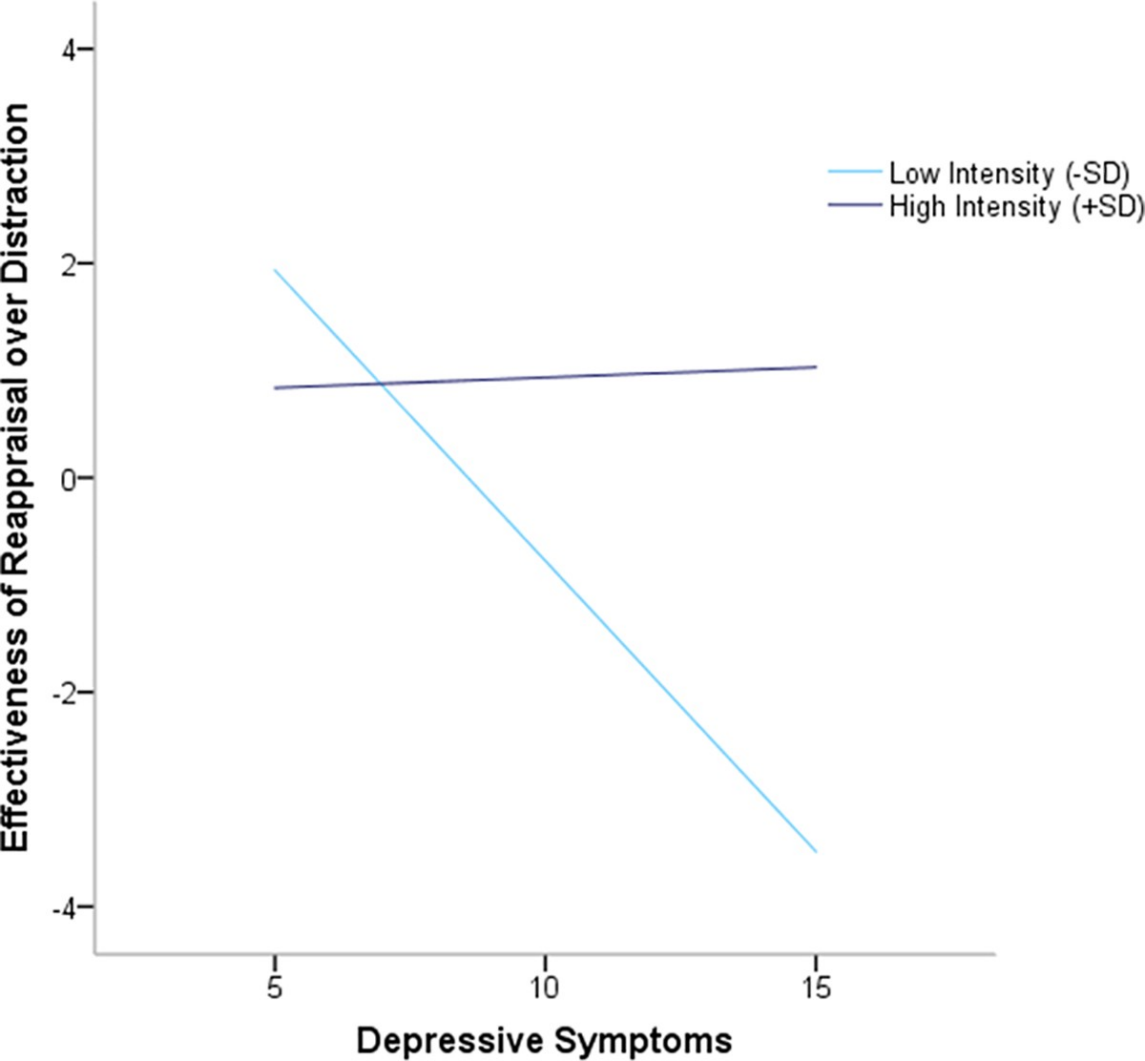
Effectiveness of regulatory strategies across a range (centered) of depressive symptoms as a function of perceived intensity levels (Experiment 2, N = 113).

In order to test this interactive effect, we conducted a moderation model in which depressive symptoms, the level of perceived intensity, and the relative effectiveness of reappraisal compared to distraction were treated as independent, moderator, and outcome variables, respectively. The general model was significant *R*^*2*^ = .18, *F*(3, 109) *=* 8.04, *p* < .001. The results of the analyses are presented in Table 4. As can be seen, there was a significant main effect of perceived intensity level, revealing that the relative effectiveness of reappraisal increased as the perceived intensity level increased. Interestingly, there was a significant main effect of depressive symptoms, indicating that reappraisal was less effective than distraction as the level of depressive symptoms increased. Most importantly, there was a significant interaction between perceived intensity level and depressive symptoms (Fig 2). In order to interpret the nature of this interaction, we computed bootstrapping confidence intervals (95%), evaluating the magnitude of the relationship between depressive symptoms and the relative effectiveness of reappraisal compared to distraction in low and high intensity levels conditions (-/+1SD). The results revealed that in low-intensity conditions, individuals with greater depressive symptoms found distraction to be more effective than reappraisal *t*(112) = -4.62, *p* < .001, while in high-intensity conditions, no such relationship was found *t*(112) = 0.24, *p* = .81. This effect accounted for an additional 4.2% of the variance, above and beyond the variance explained by the main effects.

**Table 4:** Model of the relative effectiveness of reappraisal and distraction (estimated coefficients, standard errors, and 95% confidence intervals for independent and moderator variables in Experiment 2).

Variables	*B*	*SE*	*t- value*	95% confidence interval	
				Low	High
Predictors					
Depressive symptoms***	-.20	.05	-3.62	-.31	-.09
Perceived intensity level**	.01	.0024	2.55	.001	.01
Depressive symptoms x Perceived intensity level***	.002	.0004	4.05	.001	.002

**p < .01

***p < .001.

## General discussion

The aim of the present study was twofold: first, to compare strategies preferences in conditions of low and high intensity and their actual efficacy; second, to assess the relationship between depressive symptoms and strategies’ efficacy and the moderating role of intensity levels in this relationship. When testing preferences, our results replicated previous findings, showing that individuals prefer to apply distraction in high-intensity conditions and reappraisal in low-intensity conditions [6, 26, 29, 55]. However, and most importantly, in contrast to our prediction, there were no differences in the efficacy of distraction and reappraisal as a function of intensity level. Instead, reappraisal better reduced distress in both low- and high-intensity conditions. Taken together, these results reveal a discrepancy between individuals’ regulatory preferences and the effectiveness of these preferences in reducing distress.

A possible explanation for such a discrepancy relates to the notion of suboptimal decision-making. This behavior is often expressed in making decisions that do not maximize utility or pleasure and acting in a way that does not necessarily serve us in a given situation [56–58]. Accordingly, it seems that although most individuals would prefer to divert their attention from high-intensity aversive incidents, this reaction will not ultimately promote successful emotion regulation. Support for this finding can be found in studies that have compared short- and long-term strategy effectiveness in reducing distress. These studies reveal that distraction may provide immediate relief, whereas reappraisal involves generating new meanings and interpretations that make sense of the aversive situation and thus have long-term effectiveness [31, 59]. The results contradict the process-specific timing hypothesis [24, 25], suggesting that the effectiveness of distraction and reappraisal in reducing distress does not change as a function of the intensity level of the aversive condition.

Another factor that may influence the efficacy of the emotion regulation process is the psychological state of the individual [13, 18–20, 60, 61]. Indeed, our findings demonstrate an association between depressive symptoms and the effectiveness of distraction and reappraisal, showing that while individuals with lower levels of depression symptoms benefit more from reappraisal, those with higher levels of depression benefit more from distraction.

Most importantly, we found an interactive effect of intensity and depressive symptoms on the effectiveness of distraction compared to reappraisal. Specifically, in conditions of low, but not high intensity, individuals with greater depressive symptoms found distraction to be more effective than reappraisal. This implies that the benefits of reappraisal over distraction, which has been observed among individuals from the general population, may not apply to those who exhibit higher levels of depressive symptoms in low-intensity aversive conditions.

These findings accord with the negative potentiation hypothesis, suggesting that individuals with depressive symptoms may benefit more from disengagement-related strategies, such as distraction, in low intensity conditions. In such conditions, distraction can break the cycle of negative thoughts or excessive focus on adverse aspects of the event. Possible support for this finding may rest in studies on individuals with depressive symptoms who experience immediate relief and short-term benefits in reducing distress following avoidance or distraction [31, 44, 59, 62].

The lack of significant differences in strategy effectiveness in high-intensity conditions suggests that for individuals with depressive symptoms, both reappraisal and distraction are less beneficial in reducing distress. It is possible that the high-intensity level of these conditions impairs the ability of individuals with different levels of depressive symptoms to adaptively regulate their emotions. However, future studies should further test this possibility to confirm it, while applying a gradual increase in the intensity level of these images. This may allow for an assessment of whether and at which point the intensity level disrupts the emotion regulation process.

The tension between the advantage of distraction over reappraisal in reducing distress in low-intensity conditions, and the reported preference for using reappraisal in these conditions, may shed light on the etiology and maintenance of depressive symptoms. Specifically, although speculative, it is possible that for individuals with higher depressive symptom levels, the tendency to implement reappraisal would be especially deleterious. Such implementation–which involves processing the aversive stimuli to alter its meaning–may lead to negative mood and to both the emergence and maintenance of symptoms [19, 44].

This study has several possible theoretical and clinical implications. First, the discrepancy between personal preferences and the actual value of each strategy may explain the benefit of interpersonal emotion regulation. Specifically, recent studies have revealed that applying a regulatory strategy chosen by a romantic partner, based on his or her outside perspective, helps reduce distress more effectively than applying a self-chosen strategy [63–65]. Moreover, our results imply that this evidence is especially relevant for individuals with higher levels of depressive symptoms. Specifically, whereas their ability to implement distraction may prove sufficient in downregulating distress, it is their presumable tendency to prefer reappraisal in low-intensity conditions which may mostly impair the emotion regulation process. Hence, it may be particularly important to teach individuals with higher levels of depressive symptoms how to act against their nature in order to reduce levels of distress in aversive conditions. This may be especially relevant given their tendency to constantly ruminate about daily aversive events [38], which could be reduced by adaptively using distraction. Future studies may wish to shed further light on the relationship between depression and strategy preferences by testing the effectiveness of interpersonal emotion regulation in reducing depressive symptoms. This may be done while focusing not only on the difference in effectiveness between self- and interpersonal regulation but also on the question of what guides individuals when choosing successful regulatory strategies for themselves and for others and how to encourage such adaptive behavior.

In addition, the results emphasize the necessity of carefully examining failures in different stages of the emotion regulation process. Specifically, while most studies do not differentiate between strategy preferences and their actual success in down-regulating distress [29], the current study suggests that for individuals with depressive symptoms, distraction is increasingly effective in low-intensity conditions.

The study has several limitations. First, participants’ reports of distress levels in the performance-based paradigm could have been influenced by the popular perception that reappraisal is preferable to distraction to mitigate distress [12, 14, 66, 67]. Hence, participants may have been quicker to note relief from distress following reappraisal. However, this is a general bias that may affect performances in any paradigm that involves individuals’ choices [6, 29, 68, 69]. Moreover, a performance-based paradigm is notably less subject to biases relative to questionnaires, which are widely used in studies of this nature [70, 71]. In addition, in order to continue refining their accuracy, future studies may consider monitoring physical responses such as blood pressure, muscle tension or galvanic skin response (GSR) when seeking to measure distress following emotion regulation attempts [72, 73]. Relatedly, future studies may wish to compare the effectiveness of different strategies in conditions of high and low intensity by using recordings of participants’ emotion regulation attempts [74, 75] or by directly asking participants about their perceived effectiveness. However, the latter approach requires a higher level of introspection and may reflect a biased subjective response.

Second, the study focused on individuals who display sub-clinical levels of depression, and its implications are limited to this population. This allows for the detection of more widespread trends and reflects a more recent multi-faceted framework for mental health [76–78]. Future studies may aim to compare not only the effectiveness of distraction and reappraisal in reducing distress in individuals diagnosed with major depressive disorder (MDD) but also the effectiveness of other disengagement (e.g., suppression) and engagement (e.g., rumination) strategies.

In addition, the cross-sectional design of the study does not allow for conclusions regarding causal associations between impaired emotion regulation and depressive symptoms. Further research should utilize a longitudinal design to provide a more nuanced understanding of how emotion regulation and depressive symptoms interact over time.

Lastly, an experiment conducted under laboratory conditions may not accurately reflect how emotion regulation strategies are implemented in real life. To provide a more comprehensive understanding of the process of emotion regulation, further research may choose to supplement laboratory experiments with an ecological momentary assessment (EMA) [79–81]. This could clarify how momentary emotions and thoughts interact during emotion regulation and shed light on the role of intensity and depressive symptoms in both pre-and post-regulation processes.

## Conclusions

In conclusion, the current study questions the tendency to lean on preferences in predicting the effectiveness of different regulatory strategies. This is especially important in individuals with elevated depressive symptoms, for whom the gap between preferences and actual efficacy may not only be significantly wider but can also advance the development of intrusive symptoms. Our findings offer a means to narrow this gap by encouraging psychological therapies for depression to facilitate a voluntary use of disengagement skills that can help provide short-term relief in low-intensity conditions. This could be particularly valuable in controlling rumination and preventing aversive thoughts from being deeply processed and may gradually reduce depressive symptoms.

## Supporting information

S1 Dataset S1 is Supporting information including dataset used in Experiment 1A. (XLSX)

S2 Dataset S2 is Supporting information including dataset used in Experiment 1B. (XLSX)

S3 Dataset S3 is Supporting information including dataset used in Experiment 2. (XLSX)
